# Malaria in patients with sickle cell anaemia: burden, risk factors and outcome at the Laquintinie hospital, Cameroon

**DOI:** 10.1186/s12879-019-4757-x

**Published:** 2020-01-14

**Authors:** Ngo Linwa Esther Eleonore, Samuel Nambile Cumber, Eposse Ekoube Charlotte, Esuh Esong Lucas, Mandeng Ma Linwa Edgar, Claude Ngwayu Nkfusai, Meh Martin Geh, Budzi Michael Ngenge, Fala Bede, Nzozone Henry Fomukong, Henri Lucien Fouammo Kamga, Dora Mbanya

**Affiliations:** 1 Faculty of Health Sciences, University of Bamenda, Bamenda, Cameroon; 2 Institute of Medicine, Department of Public Health and Community Medicine (EPSO), University of Gothenburg, Box 414, SE - 405 30, Gothenburg, Sweden; 3 Faculty of Health Sciences, University of the Free State, Bloemfontein, 9301 South Africa; 4 School of Health Systems and Public Health, Faculty of Health Sciences, University of Pretoria, Private Bag X323, GezinaPretoria, 0001, Pretoria, South Africa; 5 Faculty of Medicine and Pharmaceutical sciences, Douala, Cameroon; 6 Pediatric Department, Laquintinie Hospital Douala, Douala, Cameroon; 7 Faculty of Health Sciences, University of Buea, Buea, Cameroon; 8 Cameroon Baptist Convention Health Services, Bamenda, Cameroon; 9 St Mary Soledad Catholic Hospital Bamenda, Bamenda, Cameroon; 10 Etougebe Baptist Hospital, Yaounde, Cameroon

**Keywords:** Malaria in patients with sickle cell anaemia: burden, Risk factors and outcome at the laquitinie hospital

## Abstract

**Background:**

It is believed that the current prevalence of malaria in endemic areas reflects selection for the carrier form of sickle cell trait through a survival advantage. Malaria has been incriminated as a great cause of mortality in people with sickle cell disease (SCD). However, people with SCD, a high-risk group, do not benefit from free or subsisized malaria prevention and treatment in Cameroon unlike other vulnerable groups which may be due to insufficient evidence to guide policy makers. This study aimed at describing clinical and socio-demographic characteristics of patients with malaria, determining the prevalence of malaria in hospitalized children and in those with SCD and without, compare frequency of presentation of malaria related complications (using clinical and laboratory elements that define severe malaria) between children admitted for malaria with SCD and those without and finally, determing the risk factors for death in children admitted for malaria.

**Methods:**

This was a retrospective analysis of admission records of children age 1 to 18 years with a confirmed malaria diagnosis admitted at the Laquintinie Hospital during January 2015 through December 2018. Clinical features, laboratory characteristics and outcome of malarial infections, stratified by SCD status were studied. Patients with HIV infection, malnutrition, renal failure and discharged against medical advice were excluded from the study. Data were analysed using Epi-info 7 software and analysis done. Chi square test, Odds ratios, CI and student’s t test were used to determine association between variables. Statistical significance was set at *p*-value ≤0.05.

**Results:**

The prevalence of malaria was lower among children with SCD than it was among children without SCD (23.5% vs 44.9%). Similarly, among those with a positive microscopy, the mean parasite density was significantly lower among children with SCD than it was among children without SCD (22,875.6 vs 57,053.6 parasites/ μl with t-value − 3.2, *p*-value 0.002). The mean hemoglobin concentration was lower in SCD as compared to non SCD (5.7 g/l vs 7.4 g/l, t-value − 12.5, *p*-value < 0.001). Overall mortality in SCD was 3.4% and malaria was reponsible for 20.4% of these deaths as compared to the 35.4% in non SCD patients. Convulsion and impaired consciousness were significantly lower in SCD group (OR:0.1, CI: 0.1–0.3, *p* value < 0.01 and OR:0.1, CI:0.1–0.2, *p*-value < 0.001 respectively). Death was significantly higher in SCD patients with malaria as compared to SCD patients admitted for other pathologies (3.2% vs 1.5%., OR:2.2, CI:1–5, *p*-value 0.050).

**Conclusion:**

The SCD population has a lower mortality related to malaria compared to the non-SCD population. Meanwhile, within the SCD population, those admitted with malaria are twice more likely to die than those admitted for other pathologies. Jaundice, hepatomegaly and splenomegaly were common in SCD with malaria, however no risk factors for malaria severity or malaria related death was identified.

## Background

According to WHO, worldwide there was an estimated 219 million malaria cases and 435,000 deaths attributed to malaria in 2017. Approximately 41% of Cameroon population has at least 1 episode a year, with a prevalence of 29% and has an overall mortality of 30–35% [1, 2]. Despite the fact that malaria has lost its crown as the first cause of mortality in Cameroon, it is still a major cause of morbidity and mortality especially in children accounting for 67% of childhood mortality [2]. In sub-Saharan Africa, malaria claims the life of a child every twominutes [3]. It is proposed that hemoglobinopathies protect from severe life-threatening manifestation of malaria [4]. The most important of which is the mutation that causes sickle cell disease (SCD) which leads to a 90% risk reduction of severe *Plasmodium falciparum* malaria in sub-Saharan African children [5]. Sickle cell disease is an inherited chronic hematological disorder, where a point mutation in the β globin gene resulting in substitution of glutamic acid with valine at position 6 of the peptide [6, 7]. SCD is a serious public health concern, present mainly in tropical countries, especially sub-Saharan Africa [8, 9]. The World health organization (WHO) estimates that 300,000 children are born with SCD each year, 75% of whom are in sub-Saharan Africa [10, 11]. Cameroon has a sickle cell trait (SCT) prevalence of 18.2% (ranges from 8 to 34%) and a SCD prevalence of 2–3% [12–14]. The gene does not protect against infection by the malaria parasite, but it prevents establishment of disease following infection [15]. As SCT offers relative protection against malaria, one might expect the protection to be at least as effective in the homozygous state (SS) [16]. However, cinical experiences have shown it to be more dangerous since malaria does not only worsen the preexisting anemia in patients with SCD, to the point of becoming life threatening but also, the abnormal splenic function in SCD patients hinders clearance of parasitized RBCs [17]. In Africa, malaria contributes substantially to the early mortality in patients with SCD [8]. Despite this, in many countries and in Cameroon in particular, national malaria control programs do not consider this populationas a population to be at high risk of severe malaria disease. Thus, they do not benefit from free malaria treatment instituted by the program for high risk and vulnerable population as a means to tackle the burden of malaria in the country. The prevalence of malaria in the population of Douala is 24% and malaria represents 9% of admissions in this hospital. Sickle cell patients constitute 6% of all admissions at Laquintinie (non-published data). The goal of the study is to identify the SCD population as a population at risk of severe disease and identify the factors associated with severe disease and clinical outcome. This might serve as preliminary data which could be used for further studies with the aim to insert this population in the malaria control program.

## Methods

### Study design, period and setting

This was a hospital based retrospective cohort study. The study was conducted at the pediatric unit of the Douala Laquintinie Hospital, which is a second level hospital in which specialized care for SCD patients is offered through the “Centre de Prise en Charge Emmanuel Billong” where approximately 600 patients are being followed. The protocol used for management of severe malaria is artesunate. The pediatric emergency ward and the general pediatric ward were also included. Data from patients admitted between January 2015 to December 2018 were reviewed and analyzed.

### Participants and sampling

Sampling was convenient. We studied malaria in two different populations (SCD and non-SCD population). Patients aged 1 year to 18 years with documented malaria diagnosis were included. Patient**s** discharged against medical advice, who were malnourished, had HIV or renal failure were excluded from the study.

The minimum sample size was calculated in inference from a study carried out in Tanzania by Makani et al., malaria in sickle cell anaemia [18]. A total minimum sample size of 6385 patients was required (minimum exposed; 1277 and minimum non-exposed = 5108).

### Study procedures and variables

Recruitment was done using admission records. The designed data entry form was used to extract the sociodemographic and clinical characteristics as well as laboratory characteristics associated with malaria in both populations and non-malarial diseases only in sickle cell patients. Data was then verified to ensure non-repetitiveness of records and ultimately validated to be used for analysis. In this study, a malaria case was defined as any patient with a positive Rapid Diagnostic Test for malaria, parasites identified on microscopy and/or clinical improvement with just antimalarial treatment. Death from malaria referred to any death in a patient with a confirmed or suspected malaria diagnosis. Severe anaemia was said to be present if subjects had a hemoglobin level < 5 g/dl on full blood count. Hyperparasitemia was defined as parasite density of > 250,000 parasites/ μl and hyperleukocytosis as a white blood cell count of > 15,000/ μl on full blood count.

### Ethical considerations

Ethical approval was granted by the Institutional Review Board of the University of Bamenda and administrative approval was obtained from the director of the Laquintinie hospital,

### Data management and data analysis

A data spread sheet was created in excel Microsoft 2016 in which collected data was introduced and analysed through Epi-info version 7 software. Through cross tabulation of variables, the data was captured and assessment for frequency tabulation data was analyzed. Chi square test, Odds ratios (OR), Confidence Intervals (CI) and student’s t test were used to determine association between variables. Statistical significance was set at *p*-value ≤0.05. The results were presented in the form of frequency tables and charts.

## Results

### General characteristics of the study population

**Fig. 1:**
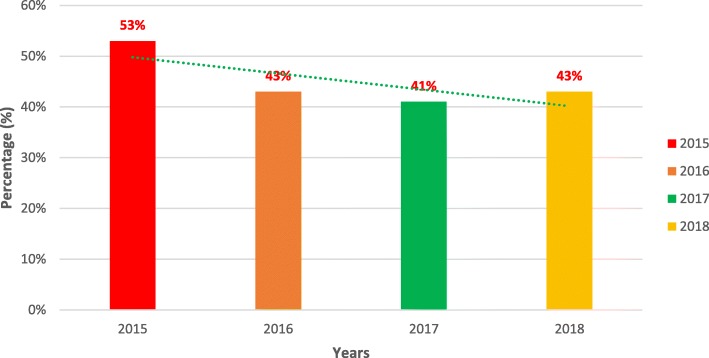
Trend in malaria admissions between the study year

A total of 6563 patients were included in the study, of which 1438 were SCD patients (22%). Of these, 982 SCD cases were admitted, with 526 (53%) being new or first-time admissions and 456 (46%) having readmissions. There was a 10% decline in malaria prevalence between 2015 and 2017 and we noticed an increase of 2% from 2017 to 2018. Fig. 1 below shows the highest prevalence of malaria was in 2015, affecting about 53% of the study population. The overall prevalence of malaria in the study was 42%, with children aged 1-5 year and males with the highest prevalence (Table 1). Among the patients admitted with sickle cell disease, Vaso-occlusive crises (not associated with infection) was the main cause of hospitalisation, accounting for 31% of admissions, followed by malaria 23%.

**Table 1:** Comparison of clinical and laboratory characteristics in children admitted with malaria with and without Sickle cell disease

Characteristics	SCD *n* = 276		Non SCD *n* = 2857		tests statistics	
	*n*	%	*n*	%	Chi-square	*p*-value
Température ≥40 °C	104	37,7%	275	9,6%	186	< 0.001*
Prostration (yes)	209	75,7%	592	20,7%	395	< 0.001*
Convulsion (yes)	10	3,6%	204	7,1%	4,89	0.03*
Hepatomegaly (yes)	74	26,8%	140	4,9%	162	< 0.001*
Splenomegaly (yes)	86	31,2%	155	5,4%	235	< 0.001*
Haemoglobinuria (yes)	9	3,3%	86	3,0%	0,054	0,817
Jaundice (yes)	46	16,7%	26	0,9%	243	< 0.001*
Respiratory distress (yes)	42	15,2%	326	11,4%	3,52	0,061
Impaired consciousness (yes)	8	2,9%	218	7,6%	8,42	0.004*
Hb level < 5 g/dL	104	37,7%	191	6,7%	280	< 0.001*
WBC > 15 (× 109 cells/L)	140	50,7%	173	6,1%	558	< 0.001*
Parasite density > 250,000 or 5%	5	1,8%	136	4,8%	5,09	0.024*
	mean	SD	mean	SD	t-value	p-value
Hb level (g/dL)	5,7	2,6	7,4	1,4	17,47	< 0.001*
Leucocyte count (× 109 cells/L)	20,3	10,9	9,8	5,6	26,66	0,394
Platelet count (cells/mm3)	274,014	175,135	177,668	98,578	14,21	< 0.001*
Parasite density (parasites/uL)	54,546	13,760	88,543	27,053	20,62	< 0.001*

* statistically significant at *p* <  0.05

Values in boldface are set at less than or equal to 0.05

In this study, the sex ratio in SCD population was 1.2:1 (male: female) as compared to 1.3:1 for the non SCD population. The 1-5 years age group had the highest number of cases. Malaria was either the primary or secondary discharge diagnosis in 23% of SCD patients at admission, compared with 49% in the non SCD population.

The overall mortality in patients with SCD was 3% as compared to 9% in the non-disease population. Malaria accounted for 35.4% of mortality in the non SCD population compared to 20.4% in the SCD population. Specific malaria mortality was higher in the non-sickle cell disease population (6%) as compared to the sickle cell population (3%) (OR: 1.8, CI: 0.9–3.5, *p*-value = 0.07).

### Factors associated with malaria in SCD and non-SCD population

Hepatomegaly was found to be significantly higher in SCD population with malaria (27% Vs 5%. OR:1.9, *p*-value < 0.001). Splenomegaly and jaundice were found to be greater in the SCD population. (31% vs 5%, OR: 2.0–3.0, *p*-value < 0.001 and 14% vs 2%, OR:6.3, *p*-value < 0.001 respectively). Convulsion and impaired consciousness were significantly lower in patients with SCD (4% vs 7%, OR:0.1, *p*-value 0.030 and 3% vs 8%, OR:0.1, *p*-value 0.004 respectively). Of the patients who died, the majority were readmitted patients and non-sicklers (Table 1).

### Laboratory characteristics associated with malaria

The mean hemoglobin concentration was significantly lower among patients with SCD at admission as compared to patients without sickle cell disease (5.7 g/dL vs 7.4 g/dL, t-value − 12.5 and *p*-value< 0.001). More SCD patients had severe anaemia compared to those without (32% Vs 18%, OR: 0.9 and *p*-value < 0.001). The mean platelet count was significantly higher in the SCD patients as compared to non SCD patient (274,014 vs 177,668 10^9^/L; t-value 14.21, *p*–value< 0.001). The mean white blood cell count was lower in patients with SCD relative to patients without SCD (20.3 vs 9.8 × 10^9^/L) though this difference was not statistically significant (t-value 26.7, *p*-value 0.394). Hyperleucocytosis was more prevalence among the SCD group than in non SCD group (43 vs 16%, OR: 5.7, *p*-value< 0.001). The mean parasite density was found to be statistically significantly lower in the SCD group than in the group without SCD (54,546 vs 88,543/ ul; t-value 20.6, *p*-value < 0.001). None of the clinical features nor laboratory findings associated with severe malaria was found to be significantly associated with SCD.

### Selected clinical and laboratory features in SCD patients with and without malaria

Temperature > 40 °C (9.6% vs 37.7%), prostration (20,7% vs 75.7%), (Jaundice (0.9% vs 16.7%, *p*-value < 0.001), splenomegaly (5.4% vs 31.2%, *p*-value < 0.001), hepatomegaly (4.9% vs 26.8%, *p*–value < 0.001), were found to be significantly associated with SCD patients with malaria (Table 2).

**Table 2:** Univariate and multivariate logistic regression analyses to show the relationship between characteristics of children hospitalised for malaria and the outcome of interest

Characteristic	Death, *n* = 176	Univariate Logistic regression		Multivariate Logistic regression	
		OR (95% CI)	*p-Value*	Adj. OR (95% CI)	*p-Value*
Gender					
Male	74 (42.1%)	1.07 (0.79–1.46)	*0,651*	1.09 (0.79–1.51)	*0.574*
Female	102 (57.9%)	1		1	
Age group					
1-5 years	110 (62.5%)	0.50 (0.17–1.45)	*0.122*¥	Variable not used in multivariate regression	
6-10 years	44 (25.0%)	0.48 (0.17–1.28)			
11-15 years	17 (9.7%)	0.38 (0.14–0.98)			
> 15 years	5 (2.8%)	1			
Year of admission					
2018	33 (19.5%)	0.77 (0.49–1.22)	*0.503*¥	Variable not used in multivariate regression	
2017	47 (27.8%)	0.91 (0.60–1.39)			
2016	42 (24.9%)	1.10 (0.72–1.69)			
2015	47 (27.8%)	1			
Season of year					
Wet	99 (66.0%)	1.12 (0.79–1.59)	*0,512*	Variable not used in multivariate regression	
Dry	51 (34.0%)	1			
Co-Morbidities					
Yes	123 (69.9%)	1.29 (0.93–1.79)	*0,132*	1.14 (0.81–1.62)	*0.446*
No	53 (30.1%)	1		1	
HIV Status					
Positive	1 (0.6%)	0.72 (0.96–5.33)	*0,745*	1.00 (0.13–7.61)	*0.996*
Negative	175 (99.4%	1		1	
Malnutrition					
Yes	2 (1.1%)	0.77 (0.18–3.19)	*0,714*	1.09 (0.26–4.60)	*0.911*
No	174 (98.9%)	1		1	
Readmission(s)*					
Yes	25 (14.2%)	4.08 (2.57–6.49)	< 0.001*	1.33 (0.77–2.29)	*0.309*
No	151 (85.8%)	1		1	
Sicke Cell Disease (SCD)*					
Yes	65 (36.9%)	7.69 (5.49–10.78)	< 0.001*	9.18 (6.12–13.78)	< 0.001*
No	111 (63.1%)	1		1	

¥ pValue reported following Wald’s test

* statistically significant at *p* < 0.05

OR is the crude Odd’s ration

Adj. OR is the adjusted Odd’s ratio

The mean hemoglobin concentration was lower in SCD patients with malaria as compared to those admitted without malaria (5.71 Vs 7.4 g/dl, *p*-value < 0.001). The mean platelet count was significantly lower in the SCD patients as compared to those without malaria (274,014/μL Vs 323,551/ μL;, *p*-value< 0.001). SCD patients with malaria had a higher risk of dying compared to those admitted for other pathologies (odds ratio 2.2, *p*-value 0.05) as represented in Table 3.

**Table 3:** Selected clinical characteristics and laboratory finding in SCD with and without malaria

	Malaria cases in SCD population	Non malaria cases in SCD population	OR	CI	*P*
Coca-Cola urine	9 (3%)	19 (2%)	0.9	0.4–2	0.834
Icteric sclerae	46 (14%)	75 (9%)	0.7	0.4–0.9	0.033
Hepatomegaly	74 (22%)	128 (16%)	0.7	0.5–0.9	**0.022**
Splenomegaly	86 (25%)	136 (17%)	0.6	0.4–0.8	**< 0.001**
Death	11 (3%)	12 (1%)	2.2	1–5	**0.050**
Mean hemoglobin (g/dL)	5.71	6.27	−4.5	–	**< 0.001**
Mean WBC count (× 10^9^ cells/L)	20.29	22.27	−2	–	0.053
Mean Platelet (cells/mm^3^)	274,014.25	323,551.3	−3.7	–	**< 0.001**

*SCD* Sickle Cell Disease, *CI* Confidence Interval, *S* Significant, *OR* Odds ratio, *WBC* white blood cell, *L* litre, *g/dL* grams per decilitre

## Discussion

Children with SCD in sub Saharan Africa are presumed to be at increased risk of malaria morbidity and mortality. This study aimed at evalutating differences between hospitalised children with SCD and those without SCD using selected clinical and para-clinical factors associated with malaria through retrospective review of hospital records. In doing so it was hoped to demonstrate the increased burden children with SCD present with and therefore the need to include SCD as a criterion to benefit from free malaria treatment.

We had a total of 6563 admissions of which 1438 admissions were SCD patients. In our study the male to female ratio of SCD was 1.20. This compares to a sex ratio of 1.37 by Mbassi Awa et al. in Yaounde (Cameroon) but less than 3.2 obtained Nansseu et al., [19, 20]. Meanwhile studies by Chemegni et al. and Aninagyei et al. found ratios of 0.96 and 0.8 respectively [21, 22]. This implies males and females should benefit from prevention programmes equally.

The prevalence of SCD was 16.4%. This was considerably higher than other studies carried out by Purohit et al. (8.95%), Komba et al. (1.6%), and Ngolet et al. (10.64%) [23–25]. The higher SCD prevalence in our study might be explained by the fact that most SCD patients living in Douala are followed in the Laquintinie hospital and thus they usually seek primary care in this health institution as compared to the non SCD population who turn to seek primary care in lower level health centers first.

Vaso-occlusive crisis was the major cause of admissions, accounting for 31.6% of all hospital admission, and malaria the second with 23.5%. This could be compared to the findings of Mbassi et al., Ngolet et al., and Aluoch et al. who had malaria prevalences of 25.5, 29.8, and 20% respectively [19, 25, 26]. This finding was lower than that found by Diop et al., Abhulimhen-Iyoha et al. who found a prevalence of 47 and 56.2% respectively [27, 28]. This might be explained by the fact that Nigeria has a very high prevalence of malaria accounting for 25% of global burden. A lower prevalence was reported by Douamba et al. (16.5%) and Aninagyei E et al. (13.4%) [22, 29]. This might be explained by the fact that only patients with parasitemia were considered to have malaria.

The overall mortality in the sickle cell population was 3.4% and of the patients who died, the highest number of deaths occurred in the 1-5 yr age group. Malaria accounted for 20.4% of these deaths, this finding is similar to the 19.6% deaths from malaria found by Purohit et al. and 16.0% found by Komba et al. [23, 24]. Two out of every 10 sickle cell patient who died had malaria. This is an important indicator that malaria affects many SCD patients and should be targeted accordingly.

We found that impaired consciousness and convulsions were lower among the SCD population as compared to the non SCD population. This was similar to findings obtained of Komba et al. and Makani [18, 24]. This can be explained by the fact that individuals with sickle cell mutation have lower adhesive properties therefore are less susceptible to damages which occur on the vascular endothelium in the pathogenesis of cerebral malaria. The lack of convulsions in this population therefore doesn’t necessarily imply less severe disease.

Splenomegaly, hepatomegaly and icteric sclerae were all higher among the SCD population as compared to those without sickle cell. Studies of Komba et al. and Makani report similar findings [18, 24]. This is thought to be due to the chronic hemolysis which characterises this condition. This argument was supported by the finding of Makani [18] who found no statistically significant difference between hepatomegaly, splenomegaly and icterus between SCD patients admitted with and without malaria. Of these, our study found only splenomegaly to be significantly associated with malaria in SCD population. This can be explained by the increased splenic clearance of parasitised RBCs. Generally, autosplenectomy occurs before the age of 5 due to chronic microvascular arteriolar occlusions in the spleen. As such the finding of splenomegaly in a SCD patient should generate a high index of suspicion for malaria.

We found a lower parasite density among SCD patients compared to non SCD population as similarily reported by Makani et al., Aninagyei et al., and Komba et al. [18, 22, 25]. This can be explained by the protective effect of the sickle mutation which causes increased clearance of parasite and the hypoxia in the RBCs which constitute an inhospitable environment for the growth of the parasite. Therefore, criteria like hyperparasitemia may not be sufficient in this population to categorize severity of disease.

Severe malaria anaemia was higher among patients with SCD as equally demonstrated by Komba et al., Makani et al. and Aninayei et al. (as shown by either a low mean hemoglobin or hemoglobin < 5 g/dl) [18, 22, 25]. As all infections in the sickle cell population, malaria worsens the preexisting anemia. Malaria was found to be the cause of most severe anaemia in hospitalized patients as demonstrated by Sumbele et al. [30]. In our study, SCD patients with malaria had a lower mean hemoglobin level than in SCD patients without malaria similar to findings by McAuley et al. [31]. Though some authors disagree with these findings [32], most agree that prevention of malaria may be useful in reducing the deleterious effect of malaria on haemoglobin and therefore reducing need for transfusions and its associated risks, and also reducing death from severe anemia in SCD patients.

In our study, we found no clinical nor laboratory characteristics associated with death in SCD population with malaria when compared to non SCD patients. Komba et al. [24] found no association between malaria parasitemia and death in SCD population as compared to the non SCD population. Makani et al. [18] had similar findings for the two sub populations (SCD and non SCD), but within the SCD population there was an increased risk of death in relation to malaria parasitemia (9.5% vs 2.2%, OR:4.9, CI;1.04–23.20), *p*-value 0.04). A high risk of death in SCD patients with malaria as compared to SCD admitted for other pathologies was observed in our study and was equally statistically significant (3% vs 1%, OR:2.2, CI:1–5, *p*-value: 0.05).

## Conclusion

SCD patients in endemic regions are recommended by WHO to receive antimalarial prophylaxis [33, 34]. Malaria is the second cause of admission in the SCD population. Though this study could not conclude that malaria was associated with higher mortality in hospitalized SCD patients compared to hospitalized non-SCD patients like in other studies [18, 35], it however demonstrated that within the SCD population, those admitted for malaria were twice at risk of death. Though no specific risk factor for death was identified, it is widely known that malaria worsens anemia and hemolysis in this population [36], and as such predisposes to more adverse outcomes.

Based on the afore mentioned findings we recommend to maintain a high index of suspicion of malaria in patients with sickle cell anaemia and be prompt to care. Strategies to reduce incidence of and mortality from malaria in this sub population (risk group) should be implemented.

## Data Availability

The datasets used and/or analyzed during the current study available from the corresponding author on reasonable request.

## Abbreviations

CI: Confidence interval
HIV: Human Immunodeficiency Virus
OR: Odds ratio
RBCs: Red Blood Cells
SCD: Sickle cell disease
SCT: Sickle cell trait
SS: Homozygous sickle cell trait
WHO: World health organization
